# Effect of *Saccharomyces cerevisiae* fermentation postbiotic supplementation on metagenomics of digital dermatitis lesions in lactating Holstein cows

**DOI:** 10.1128/spectrum.00304-26

**Published:** 2026-07-21

**Authors:** Marlee Henige, Kelly Anklam, Ilkyu Yoon, Jeffrey Wheeler, George Dawson, Dörte Döpfer

**Affiliations:** 1 Department of Medical Sciences, School of Veterinary Medicine, University of Wisconsin-Madison 5228, Madison, Wisconsin, USA; 2 Diamond V 104953 , Cedar Rapids, Iowa, USA; South China University of Technology, Guangzhou, China

**Keywords:** antimicrobial resistance genes, metagenomic, dairy cattle, postbiotic, lameness

## Abstract

Digital dermatitis (DD) is the leading cause of lameness in cattle, posing major animal welfare and economic concerns. Effective prevention strategies are increasingly important given emerging antimicrobial resistance associated with common DD treatments. Supplementation with *Saccharomyces cerevisiae* fermentation postbiotics (SCFP) has been shown to enhance innate immunity and reduce DD lesion development. This study evaluated the effect of a commercial SCFP supplement on the microbial composition of DD lesions using shotgun metagenomic sequencing to characterize microbial communities and associated antimicrobial resistance genes. Beta diversity analysis revealed that stage M4 DD lesions from SCFP-supplemented cows had a trend for different microbial compositions compared with controls (*P* = 0.051). At the genus level, M2 lesions were found to have statistically significant lower abundance of the genera *Desulfovibrio*, *Pseudomonas*, *Staphylococcus*, *Anaerotignum*, *Caproicibacterium*, and *Bacteroides* in the SCFP treatment group compared with the control (*P* < 0.05). M2 lesions from the SCFP treatment group were also found to have statistically significant higher abundance of the genera *Fusobacterium*, *Citricoccus*, *Listeria*, and *Fundicoccus* as compared with the control (*P* < 0.05). M4 lesions were found to have statistically significant lower abundance of the genera *Blautia* and *Petrimonas* in the SCFP treatment group compared with the control (*P* < 0.05). At the species level, M2 lesions were found to have statistically significant lower abundance of the species *Desulfovibrio sp. G11*, *Anaerotignum sp. MB30-C6*, *Caproicibacterium argilliputei*, and *Prevotella intermedia* in the SCFP treatment group compared with the control (*P* < 0.05). M2 lesions from the SCFP treatment group were also found to have statistically significant higher abundance of the species *Fundicoccus culcitae* and *Helcococcus ovis* as compared with the control (*P* < 0.05). Metagenomic analysis identified antimicrobial resistance genes associated with multiple antibiotics commonly used for DD treatment, including tetracyclines, lincosamides, and pleuromutilins. These findings demonstrate the potential for SCFP supplementation to alter the microbial composition of DD lesions while highlighting the ongoing concerns regarding antimicrobial resistance in DD management.

**IMPORTANCE:**

Digital dermatitis (DD) causes substantial economic loss and welfare concerns in cattle production systems worldwide. Our findings show that dietary supplementation with *Saccharomyces cerevisiae* fermentation postbiotics (SCFP) has the potential to alter the microbial ecology of DD lesions. Importantly, this work identifies antimicrobial resistance genes within DD lesions, underscoring the limitations of antibiotic-based control strategies. By linking nutritional supplementation to changes in microbial communities and resistance gene profiles, this study advances understanding of non-antibiotic approaches to disease mitigation and supports the development of sustainable, microbiome-informed management practices in food animal production.

## INTRODUCTION

Digital dermatitis (DD), an infectious disease of cattle feet, is the leading cause of lameness in dairy cattle (1, 2). While predominantly associated with cattle, DD has been identified in other ungulates, such as sheep, goats, elk, and caribou (3–7). This disease poses a significant welfare concern due to the pain caused by the characteristic circumscribed, ulcerative to papillomatous lesions located on the plantar aspect of the foot on or above the coronary band between the heel bulbs (8). Consequently, DD leads to decreased production (weight gain and milk yield), increased culling rates, and additional costs associated with treatment, which result in significant economic impacts for the industry (9–13).

Early detection and prompt treatment of DD is a crucial component of herd management; however, a strong focus should also be placed on the implementation of prevention strategies to reduce the prevalence of DD in a herd (14, 15). The use of chemical footbaths and topical antibiotics are common practice within the industry (16–18). However, these compounds can lead to environmental pollution, are a risk to human health, and contribute to antimicrobial resistance (19–21). Anecdotal evidence of reduced tetracycline and oxytetracycline efficacy in response to prolonged antimicrobial exposure had been noted in the treatment of bovine DD for years (22). However, the identification of antimicrobial resistance genes conferring resistance to various antimicrobials in bovine DD lesions occurred only more recently (23). To avoid these negative consequences of more traditional prevention practices, further research is necessary to develop alternative strategies.

One promising strategy for the prevention of DD is the development and use of nutritional feed supplements to enhance the diet. *Saccharomyces cerevisiae* fermentation postbiotics (SCFP), which consist of a complex blend of bioactive metabolites, including amino acids, organic acids, polyphenols, lipids, and B vitamins, have been widely used within the dairy industry to support cattle health and production (24, 25). Studies have indicated that SCFP supplementation increases average daily gain, increases body weight, and improves milk production (26, 27). Supplementation with SCFP has also been found to enhance the immune function of cattle through immunomodulation of the inflammatory response (28–30).

The specific SCFP blend used in this study has previously demonstrated immunomodulating properties during inflammatory challenges and stressors (30–33). Our group previously assessed the effectiveness of SCFP supplementation on controlling or preventing DD in lactating dairy cows on a commercial facility. Results showed a reduction in active DD lesions of cows supplemented with SCFP when compared with control cows (34). Our group also observed reduced rates of DD lesion development in SCFP-supplemented steers experimentally inoculated with a DD lesion homogenate, highlighting the protective effect of SCFP against DD infection (35).

Despite numerous studies investigating the effect of SCFP supplementation on the prevention of DD, many questions remain regarding the effect SCFP supplementation has on the microenvironment and varied microorganism populations of DD lesions. Studies investigating the etiology of DD have identified spirochetes belonging to the genus *Treponema* to be the primary bacterial agent associated with DD lesions (36–40). Despite the predominance of *Treponema* spp. in chronic DD lesions, additional studies have identified various other genera of organisms that may play a role in DD lesion development, commonly including *Bacteroides*, *Campylobacter*, *Dichelobacter*, *Fusobacterium*, *Mycobacterium*, *Mycoplasma*, *Porphyromonas*, and *Prevotella* (38–42).

This study aimed to investigate the effect of SCFP supplementation on the microbiota of DD lesions in lactating Holstein cows. The metagenomic analysis of DD lesion biopsy samples utilized shotgun metagenomic sequencing techniques to provide a complete genomic DNA analysis for all organisms and their antimicrobial resistance factors present in the DD lesion samples. We hypothesized that SCFP supplementation would alter the abundance of microbes present in DD lesions and their antimicrobial resistance factors.

## MATERIALS AND METHODS

### Study design

This study utilized a subset of data from a study previously described by Anklam et al. (34). A brief overview of the study is provided in the following sections.

The study was conducted between June and November 2018 for a total duration of 5 months following a randomized controlled field design. The initial 2.5 months served as a baseline for monitoring cattle health and allowing adaptation to the study diets. The final 2.5 months served as the test phase.

### Experimental animals

A total of 968 lactating Holstein cows (15 primiparous and 953 multiparous) in mid-lactation were enrolled from a commercial dairy farm (Midwest, USA) milking 3,000 cows using Astronaut A4 milking systems (Lely North America, Pella, Iowa, USA). All enrolled cows were distributed among four pens, two pens per treatment group. All cows were housed in a freestall barn with sand-bedded stalls. The alleyways were scraped with an automated alley scraper system. Cows remained in their respective pens unless they were removed to be dried off, and new cows could enter after calving. All cows received hoof trimming by a trained hoof trimmer at least once every 6 months and went through a preventative footbath with 5% copper sulfate weekly.

### Treatments

Cows were randomly split into control and SCFP supplementation groups (*N* = 484 per group, two pens per group). Cows received their respective supplementation throughout the duration of the study. Investigators were blinded as to which groups received the control or SCFP supplementation. Liquid molasses containing no supplement (control) or 19 g/day/cow of NutriTek (SCFP, Diamond V, Cedar Rapids, Iowa,USA) were mixed into the partial mixed ration based on the manufacturer recommendations and group fed to the respective pens. The ingredients and chemical composition of the partial mixed ration diet are indicated in Table 1.

**TABLE 1:** Ingredient composition of the partial mixed ration^*a*^

	Percentage of dry matter	
	Control group	SCFP group^*b*^
Ingredient		
Corn silage shredlage	45.0	45.0
Alfalfa hay	4.5	4.5
Wet corn gluten feed	14.6	14.6
Ground shelled corn	8.5	8.5
Soybean meal	7.6	7.6
Canola meal	3.1	3.1
Soy hulls	3.6	3.6
Commercial AA^*c*^	2.0	2.0
Commercial premix^*d*^	4.9	4.9
Control liquid molasses	6.3	0.0
SCFP liquid molasses	0.0	6.3
Formulated nutrient		
Crude protein	16.3	16.3
Acid detergent fiber	19.6	19.6
Neutral detergent fiber	31.5	31.5
Ether extract	3.6	3.6
Starch	21.6	21.6
Sugars	6.8	6.8

^*a*^ Corn gluten pellet fed at the robotic milker.

^*b*^ SCFP, *Saccharomyces cerevisiae* fermentation postbiotic (Diamond V, Cedar Rapids, Iowa, USA).

^*c*^ Dairy Amino Shot (Synergy Feeds LLC).

^*d*^ Formulated to provide 1.1% Ca, 0.8% Na, 0.5% Mg, 1.7% K, 0.3% S, 68 mg/kg Zn, 61 mg/kg Mn, 17 mg/kg Cu, 213 mg/kg Fe, 0.5 mg/kg I, 0.3 mg/kg Co, and 0.5 mg/ kg Se, 6,867 IU of vitamin A/kg, 1,717 IU of vitamin D3/kg, and 58 IU of vitamin E/kg.

### Data collection

Lame cows were identified by herdsmen during pen walks once per day. Cows exhibiting signs of lameness were separated and examined for the presence of DD lesions. All DD lesions were graded using M-stages on a five-point scale. Lesions were classified as M0 if no lesion was observed at the coronary band; M1 if a focal lesion <2 cm in diameter was observed surrounded by healthy skin; M2 if an active lesion ≥2 cm in diameter was observed; M4 if the lesion was chronic hyperkeratotic (M4) or proliferative (M4P); or M4.1 if the lesion was chronic hyperkeratotic (M4.1) or proliferative (M4.1P) combined with M1 lesions within the lesion perimeter (43, 44). The most severe DD score per two hindfeet, cow, and timepoint was recorded using the following hierarchy of severity: M0 < M1 < M4.1 < M4.1P < M4 < M4P < M2. The chronicity of each lesion was also recorded, with chronicity level 1 representing hyperkeratotic lesions and chronicity level 2 representing proliferative lesions. Active DD lesions were treated with QuickHit Gel (SSI Corporation, Julesburg, Colorado, USA), a nonantibiotic chelated copper gel under a light wrap, which was removed after 1–2 days.

### Skin biopsy collection

Biopsies of DD lesions were collected at the end of the study, 5 months post the start of control and SCFP supplementation. Using a convenience sampling approach, 20 cows (*N* = 10 per treatment group) with DD lesions were randomly selected from the four pens of cows enrolled in the study. DD lesion biopsy sample collection was performed under local anesthesia by subcutaneously administering 6 mL of 2% lidocaine hydrochloride (Sparhawk Laboratories Inc., Lenexa, KS, USA) with a 20-gauge needle. The biopsy area was rinsed with water to clean and remove any gross organic debris prior to collection. Samples were collected using a 6-mm biopsy punch. Following collection, samples were rinsed with sterile saline solution (Walgreens, Deerfield, IL, USA) and were placed in sterile 5.0-mL tubes, transported on ice, and stored at −80°C until further analysis. Table 2 is a summary of the collected DD lesion biopsy samples.

**TABLE 2:** Summary of biopsy samples of digital dermatitis (DD) lesions collected from Holstein cows supplemented with or without *Saccharomyces cerevisiae* fermentation postbiotic (SCFP)^*a*^

Sample number	Treatment group	Foot^*b*^	M-stage^*c*^	Lesion chronicity^*d*^	Pen
1	Treatment	LR	M4	2	B
2	Treatment	RR	M4	1	A
3	Treatment	RR	M4	2	A
4	Control	RR	M4	2	D
5	Control	RR	M2	2	C
6	Control	LR	M4	2	C
7	Control	RR	M2	2	C
8	Control	LR	M4	2	C
9	Control	LR	M4	2	D
10	Control	RR	M4	1	D
11	Treatment	LR	M2	1	B
12	Treatment	RR	M4	1	B
13	Treatment	LR	M4	1	B
14	Treatment	RR	M4	2	B
15	Treatment	LR	M2	1	B
16	Treatment	RR	M2	1	B
17	Treatment	RR	M4	1	B
18	Control	RR	M4	1	C
19	Control	LR	M2	2	C
20	Control	RR	M2	2	C

^*a*^ SCFP, *Saccharomyces cerevisiae* fermentation postbiotic (Diamond V, Cedar Rapids, Iowa, USA).

^*b*^ Foot: left rear (LF) or right rear (RR).

^*c*^ M-stage: an active lesion ≥2 cm in diameter (M2) or a chronic hyperkeratotic lesion without proliferation (M4).

^*d*^ Lesion chronicity: a hyperkeratotic lesion (1) or a proliferative lesion (2).

### Metagenomic shotgun sequencing analysis

Biopsy samples were processed for DNA extraction by transferring the biopsy samples to 2.0 ml PowerBead tubes (Qiagen, Valencia, California, USA) containing buffer ATL and proteinase K (DNeasy blood and tissue kit, Qiagen, Valencia, California, USA), homogenized using a BeadBlaster24 (Benchmark Scientific, Edison, New Jersey, USA), and incubated overnight at 56°C. Following overnight incubation, biopsy samples were processed as directed by the manufacturer’s recommendations (DNeasy blood and tissue kit, Qiagen, Valencia, California, USA), and sample DNA was eluted in 100-µL buffer AE. Total DNA yield was quantified with the Nanodrop 2000c spectrophotometer (Thermo Fisher Scientific, Waltham, Massachusetts, USA) and stored at −80°C prior to downstream processing (45, 46).

Whole genome shotgun sequencing was performed on each sample by the University of Wisconsin Biotechnology Center DNA Sequencing Facility using the Novaseq platform. The sequence and statistical analysis were provided by the University of Wisconsin Bioinformatics Resource Center (47–53).

### Statistical analysis

All statistical analyses were conducted using R version 4.4.3 software (54). The packages used were ANCOMBC (v2.10.1), dplyr (v1.1.4), ggh4x (v0.3.1), ggplot2 (v4.0.0), ggpubr (v0.6.1), phyloseq (v1.52.0), scales (v1.4.0), and vegan (v2.7-1) (55–64).

Alpha diversity metrics, including the Chao1, Shannon, and Inverse Simpson indices, were calculated using species level absolute count data, excluding bovine DNA, between treatment groups at the M-stage and pen levels (65–68). Statistical significance was determined using a Wilcoxon test. Beta diversity metrics were calculated using Bray-Curtis dissimilarity on relative abundance of species for each sample, excluding bovine DNA, between treatment groups at the M-stage and pen levels (69). The beta diversity dissimilarity matrix was visualized using principal coordinates analysis. Statistical significance was determined using permutational multivariate analysis of variance (PERMANOVA) and permutational multivariate analysis of dispersion (PERMDISP) tests.

To visualize the composition of the most abundant organisms present in the samples, the top 15 genera and 25 species, excluding bovine DNA, were identified from the total abundance values of all samples in the study. Relative abundance was calculated using values from the top 15 genera and 25 species for each individual sample. All other taxa were combined into a single category called “Other.” Stacked barplots were created to visualize the microbial composition at both the genus and species levels. Differential abundance analysis between absolute microbial abundances of all genera and species for each treatment group was calculated at each M-stage (M2 and M4) using analysis of compositions of microbiomes with bias correction (ANCOM-BC) (57). Given the limited sample size, statistical significance for the differential abundance was reported for both unadjusted and Benjamini-Hochberg adjusted *P* values at the 0.1 significance level to highlight potential patterns that may warrant future investigation. Benjamini-Hochberg adjustment corrects for multiple hypothesis tests being performed simultaneously to prevent increased false discovery rates, which are common in genomic analyses due to the large number of organisms being evaluated (70). Conclusions in this study were based only on results meeting the conventional 0.05 significance level.

Antimicrobial resistance genes (ARGs) were identified by aligning each DNA sequence to the comprehensive antibiotic resistance database (CARD) (71). Results were then normalized from the read counts to fragments per kilobase of transcript per million mapped reads (FPKM). Using the FPKM normalized data, a heatmap visualizing gene expression of resistance against antibiotic drugs for each biopsy sample was generated.

## RESULTS

### Microbial diversity

#### Alpha diversity

**Fig 1:**
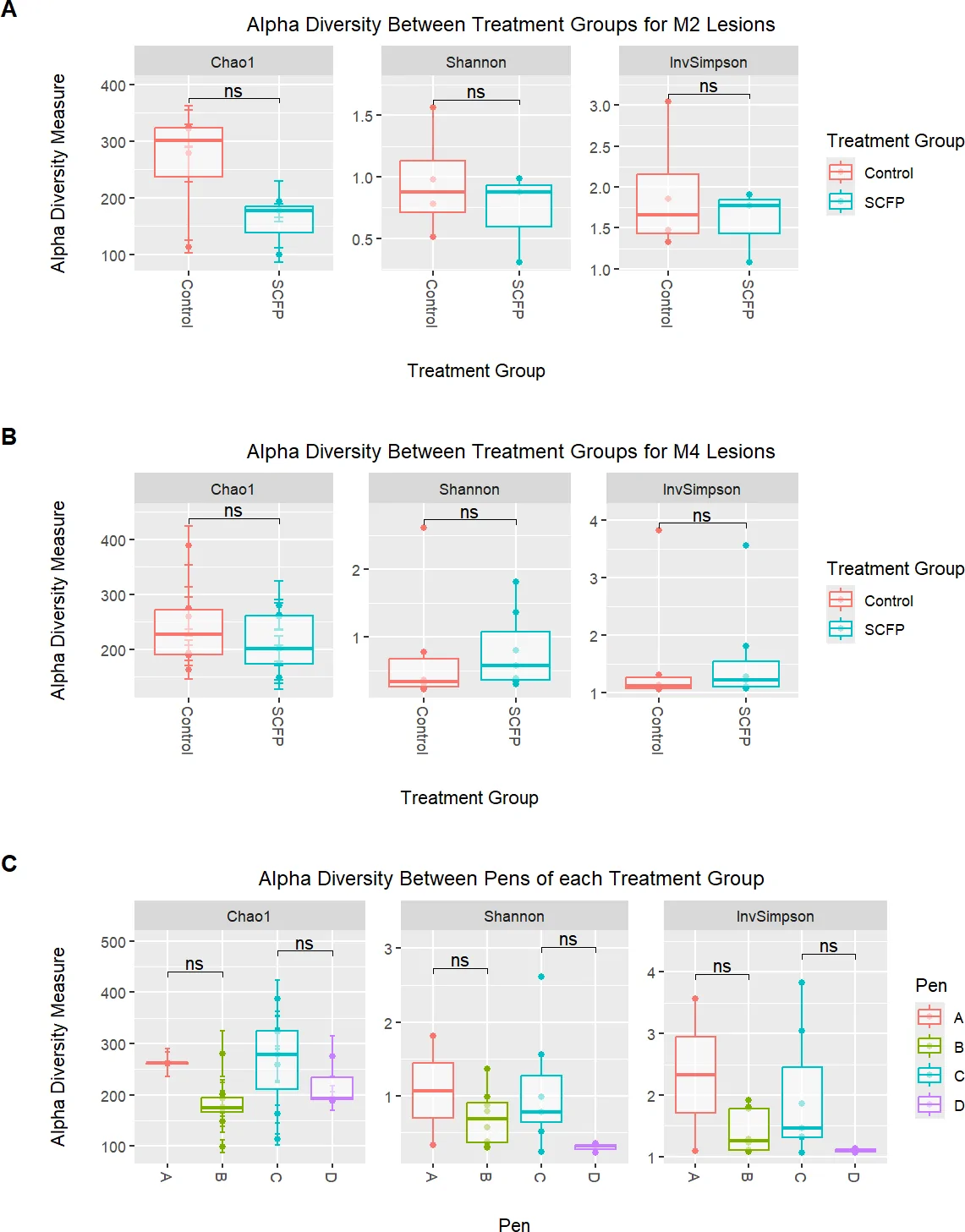
Alpha diversity metrics (Chao1, Shannon, and Inverse Simpson) for biopsies of digital dermatitis (DD) lesions from Holstein cows supplemented with or without *Saccharomyces cerevisiae* fermentation postbiotic (SCFP). (**A**) Comparison between treatment groups for M2 lesions. (**B**) Comparison between treatment groups for M4 lesions. (**C**) Comparison between pens of each treatment group. ns: not significant (*P* > 0.05).

Alpha diversity metrics were calculated using the Chao1, Shannon, and Inverse Simpson indices to analyze the richness and evenness of the microbial composition among individual digital skin biopsy samples between treatment groups at the M-stage and pen levels. Metrics were visualized using box and whisker plots as shown in Fig. 1. Results of this analysis revealed no statistical difference between treatment groups for either M2 or M4 lesions. There was also no difference between pens of each respective treatment group. This suggests that there were similar richness and evenness in samples between treatment groups of both active (M2) and chronic (M4) DD lesions.

#### Beta diversity

**Fig 2:**
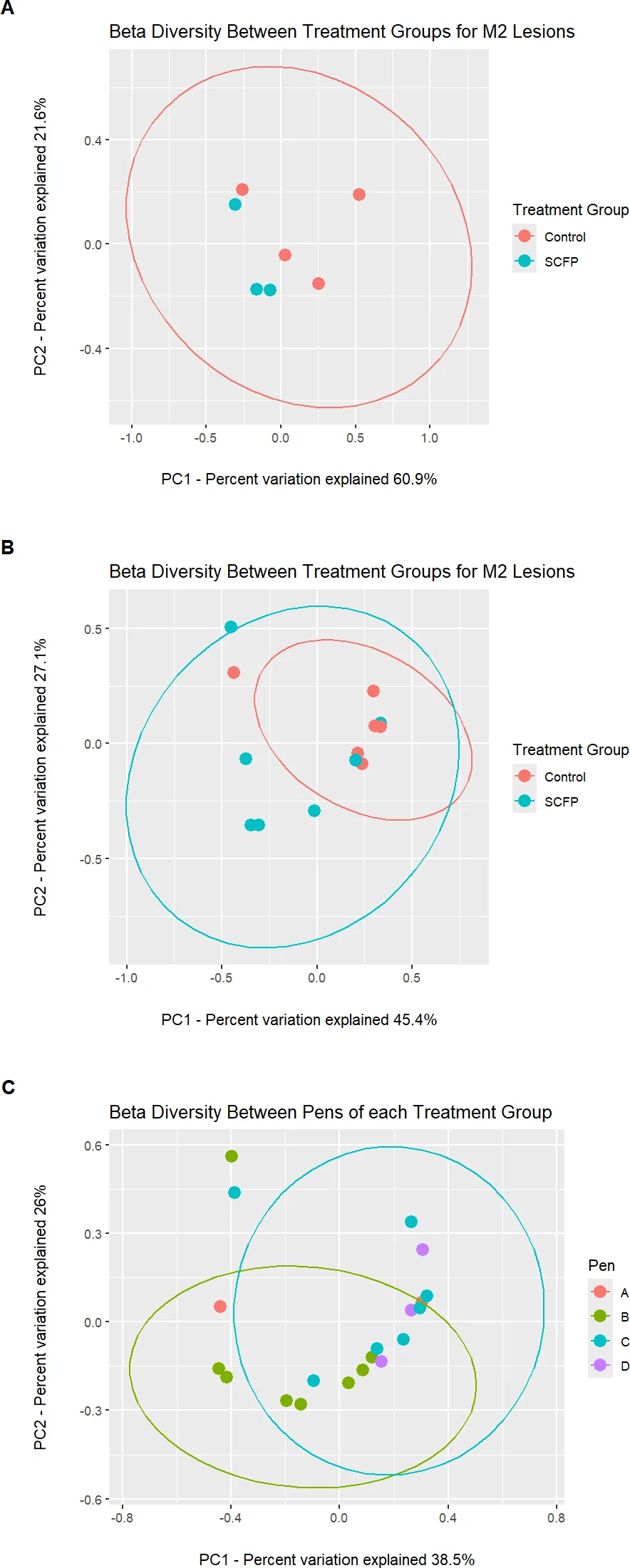
Bray-Curtis dissimilarity beta diversity metrics plotted using principal coordinates analysis for biopsies of digital dermatitis (DD) lesions from Holstein cows supplemented with or without *Saccharomyces cerevisiae* fermentation postbiotic (SCFP). (**A**) Comparison between treatment groups for M2 lesions. (**B**) Comparison between treatment groups for M4 lesions. (**C**) Comparison between pens of each treatment group.

Beta diversity metrics were calculated using Bray-Curtis dissimilarity to evaluate the microbial diversity among digital skin biopsy samples between treatment groups at the M-stage and pen levels. The dissimilarity matrices were visualized using principal coordinates analysis as shown in Fig. 2. PERMANOVA testing revealed a trend for significant difference in microbial composition between SCFP-supplemented cows and controls with M4 lesions (*P* = 0.051). There was no statistical difference between treatment groups with M2 lesions or between pens of each treatment group. PERMDISP testing revealed no differences between treatment groups with either M2 or M4 lesions and no differences between pens of each treatment group, suggesting that group dispersions were homogeneous. This indicates that the trend for difference between treatment groups with M4 lesions could be attributed to varied community structure rather than within-group variability.

### Microbial composition

#### Genus level composition

**Fig 3:**
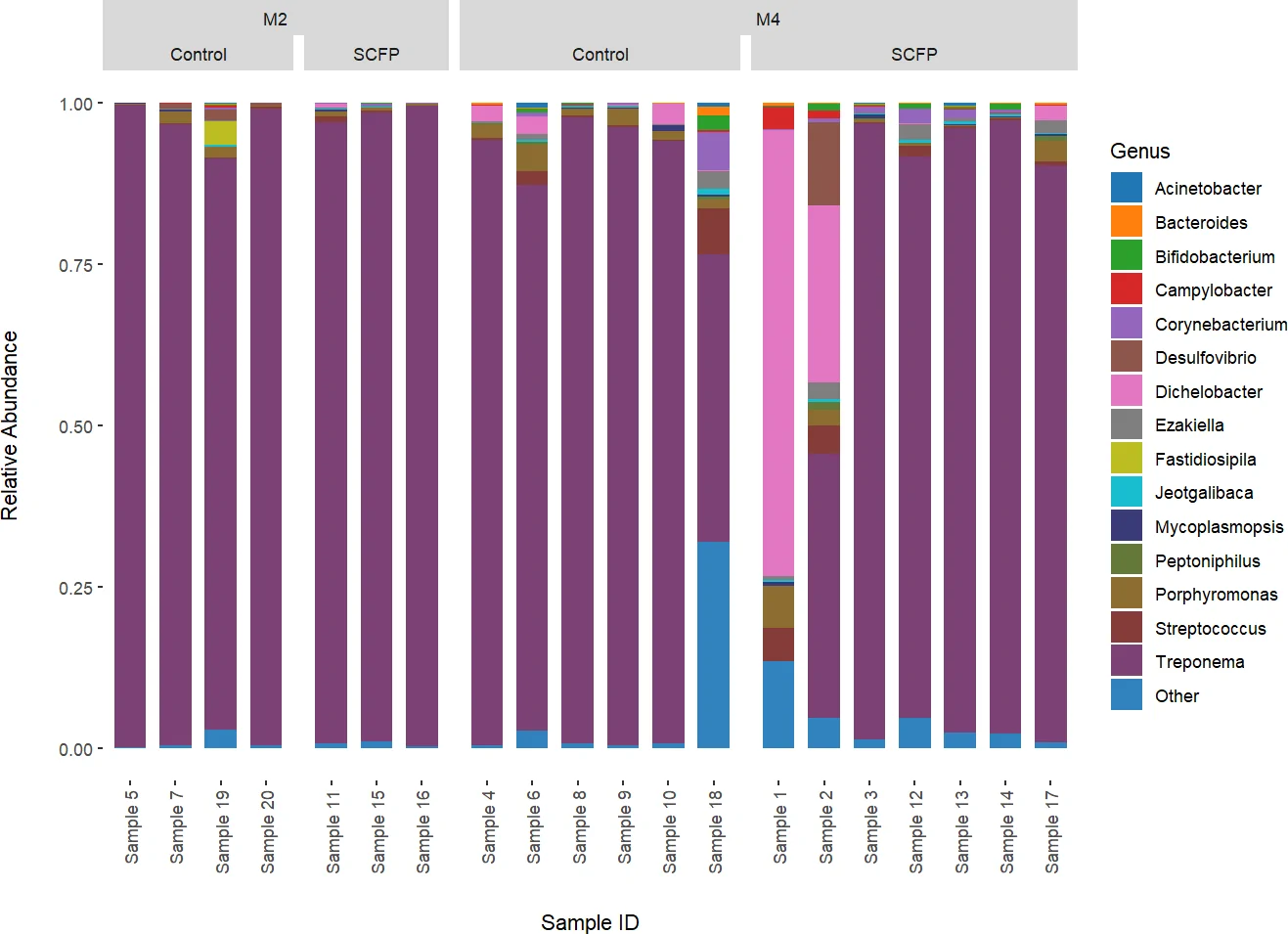
Stacked barplot depicting the relative abundance of the top 15 microbial genera for biopsy samples of digital dermatitis (DD) lesions collected from Holstein cows supplemented with or without *Saccharomyces cerevisiae* fermentation postbiotic (SCFP), faceted by treatment group and M-stage. M-stages: M2 lesion (an active lesion ≥2 cm in diameter) and M4 lesion (a chronic hyperkeratotic lesion without proliferation). *N* = 20 (*N* = 10 per treatment group).

Relative abundances of the top 15 genera for each treatment group, faceted by M-stage, were visualized in stacked barplots (Fig. 3). Genera outside the top 15 most abundant taxa were grouped into a sixteenth category labeled “Other.” A relative predominance of the *Treponema* genus was observed in the majority of samples (Fig. 3). Samples 1 and 2 had a higher relative abundance of the genus *Dichelobacter* than the other samples, whereas sample 18 had the highest relative abundance of other genera. These differences illustrated the complex and shifting microbial composition of DD lesions during progression to chronic M-stage (M4). Because no significant pen effect was detected in the alpha and beta diversity analyses, samples within each treatment group were analyzed together rather than separated by pen. Results of the associated ANCOM-BC statistical testing are shown in Tables 3 and 4, comparing treatment groups with M2 and M4 lesions respectively. Due to the limited sample size and abundance of genera present in the samples, both Benjamini-Hochberg adjusted and unadjusted *P* values were reported at the 0.1 significance level. Adjusted *P* values from the M2 lesion analysis revealed statistically significant lower abundance of the genera *Desulfovibrio*, *Pseudomonas*, *Staphylococcus*, *Anaerotignum*, *Caproicibacterium*, and *Bacteroides* in the SCFP treatment group compared with the control (*P* < 0.05). M2 lesions from the SCFP treatment group were also found to have statistically significant higher abundance of the genera *Fusobacterium*, *Citricoccus*, *Listeria*, and *Fundicoccus* as compared with the control (*P* < 0.05). Adjusted *P* values from the M4 lesion analysis revealed significantly lower abundance of the genera *Blautia* and *Petrimonas* in the SCFP treatment group compared with the control (*P* < 0.05). This suggests that SCFP supplementation may play a role in altering the presence of specific microbial genera within bovine digital skin in the case of both active (M2) and chronic (M4) DD lesions.

**TABLE 3:** Genus level results of analysis of compositions of microbiomes with bias correction (ANCOM-BC) for the microbial composition of biopsy samples of stage M2 digital dermatitis (DD) lesions collected from Holstein cows supplemented with or without *Saccharomyces cerevisiae* fermentation postbiotic (SCFP)^*a*^^,^^*b*^^,^^*c*^

Genus	Log fold change	*P* value	Adjusted *P* value
***Desulfovibrio***	−5.49211	**2.22893E-21**	**4.79220E-19**
***Fusobacterium***	0.99243	**0.00006**	**0.00246**
*Bradyrhizobium*	0.46350	0.07504	0.41370
*Porphyrobacter*	0.46350	0.07504	0.41370
***Sphingomonas***	0.69455	**0.03519**	0.26824
***Sphingobium***	0.46350	**0.01203**	0.13611
*Novosphingobium*	0.46350	0.07504	0.41370
*Variovorax*	0.46350	0.07504	0.41370
***Pseudomonas***	−0.56206	**0.00128**	**0.03048**
***Parafannyhessea***	−0.76480	**0.03618**	0.26824
***Thermomonospora***	0.59865	**0.02252**	0.19366
***Cutibacterium***	0.67916	**0.04559**	0.30633
***Dermabacter***	0.46350	**0.01203**	0.13611
***Leucobacter***	0.46350	**0.01203**	0.13611
*Agrococcus*	0.46350	0.07504	0.41370
*Microbacterium*	0.55505	0.05318	0.34648
***Citricoccus***	0.52126	**0.00167**	**0.03591**
***Kocuria***	0.46350	**0.01203**	0.13611
***Mobiluncus***	1.99820	**0.02854**	0.23004
*Trueperella*	−1.92022	0.07495	0.41370
***Dialister***	0.65642	**0.00716**	0.11841
*Erysipelothrix*	−1.20631	0.08560	0.42798
***Listeria***	0.69455	**0.00002**	**0.00118**
*Kurthia*	0.59865	0.08528	0.42798
***Staphylococcus***	−1.75696	**0.00041**	**0.01107**
*Heyndrickxia*	1.16403	0.08206	0.42798
***Fundicoccus***	0.75231	**0.00025**	**0.00912**
***Dolosigranulum***	−0.88491	**0.01640**	0.17629
***Filifactor***	−1.55173	**0.02081**	0.19366
*Alkaliphilus*	0.69455	0.09573	0.43856
***Anaerotignum***	−3.10276	**0.00002**	**0.00118**
***Mediterraneibacter***	0.59865	**0.02252**	0.19366
*Lachnoanaerobaculum*	0.69455	0.09573	0.43856
***Butyrivibrio***	−1.97379	**0.00285**	0.05578
***Caproicibacterium***	−1.08192	**4.19014E-06**	**0.00045**
*Dysosmobacter*	0.59865	0.08528	0.42798
***Mageeibacillus***	0.46350	**0.01203**	0.13611
***Tissierella***	−0.63398	**0.01914**	0.18703
***Elizabethkingia***	0.46350	**0.01203**	0.13611
***Cruoricaptor***	−1.14991	**0.04362**	0.30633
***Flavobacterium***	0.83651	**0.02,889**	0.23,004
***Petrimonas***	−0.76480	**0.00595**	0.10654
***Prevotella***	−1.04414	**0.01735**	0.17764
***Bacteroides***	−2.10643	**0.00038**	**0.01107**
***Porphyromonas***	−0.90289	**0.04504**	0.30633

^*a*^ SCFP, *Saccharomyces cerevisiae* fermentation postbiotic (Diamond V, Cedar Rapids, Iowa, USA).

^*b*^ Bolded values represent statistical significance at the 0.05 significance level (*P* < 0.05).

^*c*^ *N* = 7 (SCFP = 3; control = 4).

**TABLE 4:** Genus level results of analysis of compositions of microbiomes with bias correction (ANCOM-BC) for the microbial composition of biopsy samples of stage M4 digital dermatitis (DD) lesions collected from Holstein cows supplemented with or without *Saccharomyces cerevisiae* fermentation postbiotic (SCFP)^*a*^^,^^*b*^^,^^*c*^

Genus	Log fold change	*P* value	Adjusted *P* value
***Sphingobium***	0.64347	**0.01719**	0.40824
*Haemophilus*	0.46226	0.06353	0.53847
***Psychrobacter***	−0.80975	**0.03461**	0.46968
*Tessaracoccus*	0.42893	0.05154	0.51131
***Cutibacterium***	1.20510	**0.00379**	0.21492
*Janibacter*	0.47731	0.07881	0.54399
*Serinicoccus*	0.34641	0.09906	0.62741
*Leucobacter*	0.48685	0.08017	0.54399
***Rothia***	0.89944	**0.00452**	0.21492
***Flaviflexus***	0.99259	**0.00976**	0.30916
***Turicibacter***	0.63934	**0.04035**	0.47920
***Catenibacterium***	−0.57356	**0.03358**	0.46968
*Solobacterium*	−0.64962	0.05382	0.51131
*Gemella*	0.40433	0.07201	0.54399
***Ureibacillus***	0.32991	**0.01185**	0.32164
***Staphylococcus***	0.99585	**0.03753**	0.47539
*Alkalibacterium*	0.34641	0.05313	0.51131
*Trichococcus*	0.40433	0.06151	0.53847
*Granulicatella*	0.68376	0.06978	0.54399
***Lactococcus***	0.50335	**0.00929**	0.30916
***Coprococcus***	−0.51908	**0.03323**	0.46968
***Blautia***	−0.75666	**0.00002**	**0.00319**
***Symbiobacterium***	0.40433	**0.02802**	0.46968
***Acutalibacter***	−0.54478	**0.03282**	0.46968
*Thermoclostridium*	0.40433	0.06518	0.53847
*Flavonifractor*	−0.44889	0.07484	0.54399
*Anaerococcus*	−0.59555	0.05178	0.51131
***Sphingobacterium***	−0.61236	**0.02444**	0.46968
***Petrimonas***	−0.79546	**0.00024**	**0.02311**
*Hoylesella*	1.50430	0.09,379	0.61,450

^*a*^ SCFP, *Saccharomyces cerevisiae* fermentation postbiotic (Diamond V, Cedar Rapids, Iowa, USA).

^*b*^ Bolded values represent statistical significance at the 0.05 significance level (*P* < 0.05).

^*c*^ *N* = 13 (SCFP = 7; control = 6).

#### Species-level composition

**Fig 4:**
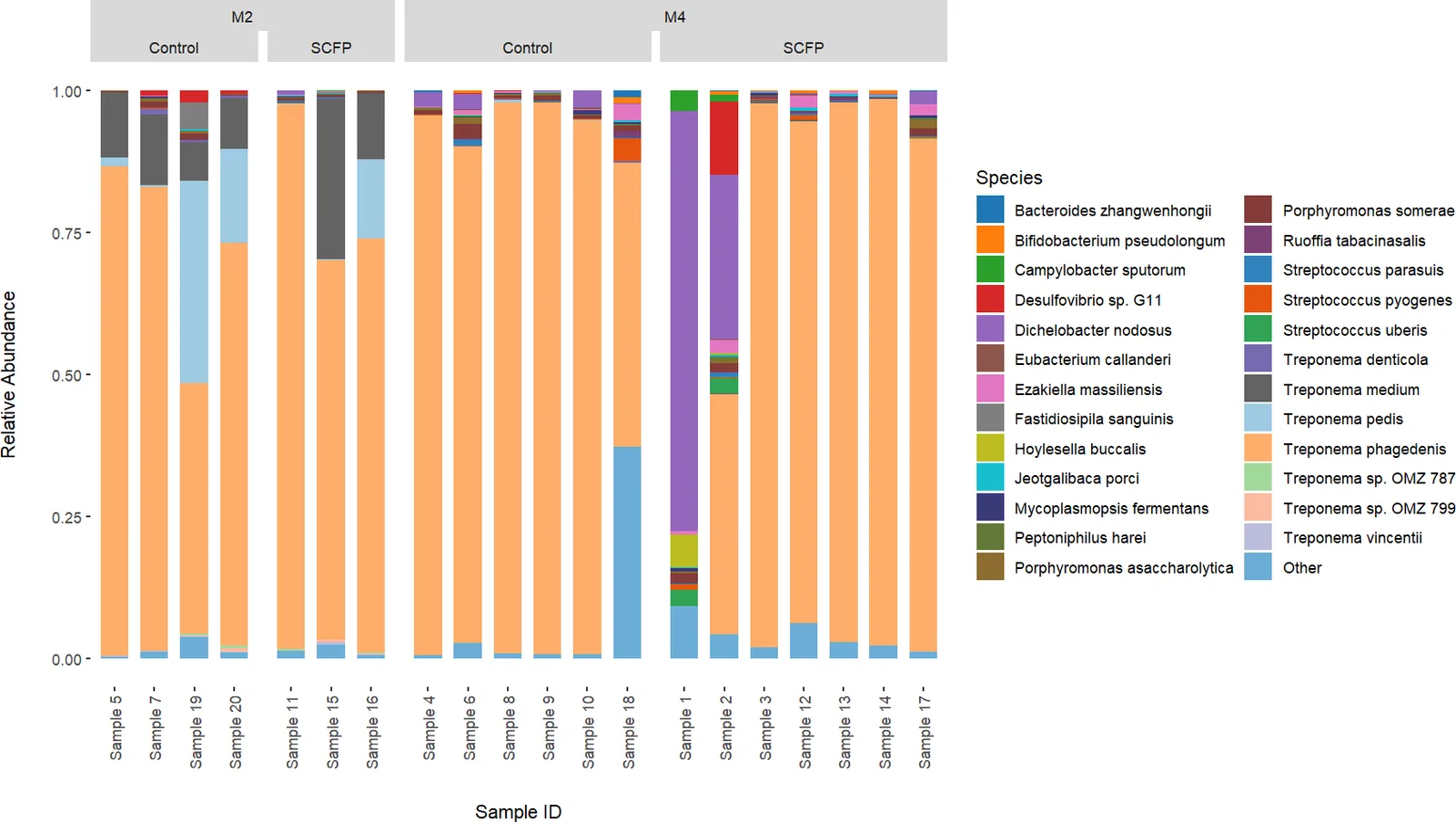
Stacked barplot depicting the relative abundance of the top 25 microbial species for biopsy samples of digital dermatitis (DD) lesions collected from Holstein cows supplemented with or without *saccharomyces cerevisiae* fermentation postbiotic (SCFP), faceted by treatment group and M-stage. M-stages: M2 lesion (an active lesion ≥2 cm in diameter) and M4 lesion (a chronic hyperkeratotic lesion without proliferation). *N* = 20 (*N* = 10 per treatment group).

Relative abundances of the top 25 species for each treatment group, faceted by M-stage, were visualized in stacked barplots (Fig. 4). Species outside the top 25 most abundant taxa were grouped into a twenty-sixth category labeled “Other.” A relative predominance of *Treponema phagedenis* was observed in the majority of samples. Samples 1 and 2 had a higher relative abundance of *Dichelobacter nodosus*, whereas sample 18 had the highest relative abundance of other species, consistent with findings from the genus level analysis above. Due to no significant pen effect found in the alpha and beta diversity analyses, samples within each treatment group were analyzed together rather than separated by pen. Results of the associated ANCOM-BC statistical testing are shown in Tables 5 and 6, comparing treatment groups with M2 and M4 lesions respectively. Due to the limited sample size and abundance of species present in the samples, both Benjamini-Hochberg adjusted and unadjusted *P* values were reported at the 0.1 significance level. Adjusted *P* values from the M2 lesion analysis revealed statistically significant lower abundance of the species *Desulfovibrio sp. G11*, *Anaerotignum sp. MB30-C6*, *Caproicibacterium argilliputei*, and *Prevotella intermedia* in the SCFP treatment group compared with the control (*P* < 0.05). M2 lesions from the SCFP treatment group were also found to have statistically significant higher abundance of the species *Fundicoccus culcitae* and *Helcococcus ovis* as compared with the control (*P* < 0.05). Adjusted *P* values from the M4 lesion analysis revealed no statistically significant differences between treatment groups. This suggests that SCFP supplementation may play a role in altering the presence of specific microbial species within bovine digital skin in the case of active (M2) DD lesions.

**TABLE 5:** Species level results of analysis of compositions of microbiomes with bias correction (ANCOM-BC) for the microbial composition of biopsy samples of stage M2 digital dermatitis (DD) lesions collected from Holstein cows supplemented with or without *saccharomyces cerevisiae* fermentation postbiotic (SCFP)^*a*^^,^^*b*^^,^^*c*^

Species	Log fold change	*P* value	Adjusted *P* value
***Desulfovibrio sp. G11***	−5.47104	**6.18257E-21**	**2.35556E-18**
*Fusobacterium animalis*	1.44090	0.06076	0.47244
*Fusobacterium necrophorum*	0.95018	**0.01567**	0.20581
*Devosia sp. MC521*	0.66306	**0.03906**	0.34979
*Porphyrobacter sp. ULC335*	0.43201	0.07816	0.48815
*Sphingomonas sp. AAP5*	0.43201	**0.00662**	0.09992
*Novosphingobium sp. THN1*	0.43201	0.07816	0.48815
*Denitrificimonas caeni*	−0.63209	0.09374	0.53468
*Parafannyhessea umbonata*	−0.79629	**0.02575**	0.27250
*Thermobifida fusca*	−0.49219	0.09314	0.53468
*Thermomonospora curvata*	0.56716	**0.02289**	0.25654
*Cutibacterium acnes*	0.67963	**0.04531**	0.38360
*Leucobacter denitrificans*	0.43201	**0.00662**	0.09992
*Agrococcus beijingensis*	0.43201	0.07816	0.48815
*Glutamicibacter protophormiae*	0.48977	0.05336	0.43944
*Mobiluncus massiliensis*	1.86142	**0.03948**	0.34979
*Bifidobacterium choerinum*	−0.54797	**0.03867**	0.34979
*Corynebacterium accolens*	0.43201	**0.00662**	0.09992
*Corynebacterium sp. Z-1*	0.43201	**0.00662**	0.09992
*Corynebacterium camporealensis*	0.43201	**0.00662**	0.09992
*Corynebacterium freneyi*	0.43201	0.07816	0.48815
*Corynebacterium kefirresidentii*	0.43201	0.07816	0.48815
*Dialister pneumosintes*	0.79821	**0.00114**	0.06186
*Erysipelothrix sp. HDW6A*	−1.13582	**0.04065**	0.35196
*Listeria monocytogenes*	0.43201	**0.00662**	0.09992
*Listeria innocua*	0.43201	0.07816	0.48815
*Ureibacillus thermophilus*	0.43201	**0.00662**	0.09992
*Heyndrickxia coagulans*	1.13254	0.09683	0.53468
***Fundicoccus culcitae***	0.72082	**0.00011**	**0.00834**
*Globicatella sp. PHS-GS-PNBC-21-1553*	−1.50295	**0.00190**	0.07242
*Aerococcus viridans*	0.43201	**0.00662**	0.09992
*Dolosigranulum pigrum*	−0.80538	**0.00805**	0.11357
*Jeotgalibaca ciconiae*	0.43201	**0.00662**	0.09992
*Streptococcus ruminantium*	0.56716	**0.02289**	0.25654
*Streptococcus alactolyticus*	0.43201	0.07816	0.48815
*Streptococcus sp. Marseille-Q6470*	0.43201	0.07816	0.48815
*Streptococcus agalactiae*	0.72082	0.08217	0.50494
*Streptococcus porcinus*	1.18405	0.08579	0.51071
*Streptococcus pneumoniae*	0.75160	0.06404	0.47843
*Filifactor alocis*	−1.58322	**0.01908**	0.24234
***Anaerotignum sp. MB30-C6***	−3.12678	**0.00001**	**0.00150**
*Hungatella hathewayi*	−0.31890	**0.03,486**	0.34058
*[Ruminococcus] lactaris*	0.56,716	**0.02,289**	0.25654
*Roseburia hominis*	0.66306	**0.03906**	0.34979
*Blautia coccoides*	0.43201	**0.00662**	0.09992
*Butyrivibrio fibrisolvens*	−2.00528	**0.00227**	0.07855
***Caproicibacterium argilliputei***	−0.94013	**2.92983E-06**	**0.00056**
*Dysosmobacter welbionis*	0.56716	0.09620	0.53468
*Mageeibacillus indolicus*	0.43201	**0.00662**	0.09992
*Clostridium intestinale*	0.56,716	**0.02289**	0.25654
*Clostridium kluyveri*	−0.78100	**0.00151**	0.07205
*Clostridium sp. BJN0013*	0.43,201	0.07816	0.48815
*Tissierella sp. MB52-C2*	−0.66548	**0.00454**	0.09992
*Anaerococcus prevotii*	0.68269	0.06345	0.47843
***Helcococcus ovis***	1.08124	**0.00014**	**0.00871**
*Helcococcus kunzii*	−1.06151	0.05421	0.43944
*Peptoniphilus genitalis*	−0.54797	**0.02438**	0.26534
*Cruoricaptor ignavus*	−1.18140	**0.02849**	0.29334
*Flavobacterium ammonificans*	0.58576	0.05607	0.44503
*Parabacteroides distasonis*	0.43201	0.07816	0.48815
***Prevotella intermedia***	−1.18336	**0.00002**	**0.00150**
*Bacteroides zhangwenhongii*	−1.43285	0.08435	0.51012
*Bacteroides thetaiotaomicron*	−1.67480	**0.00616**	0.09992
*Porphyromonas pogonae*	−1.33515	**0.00459**	0.09992
*Porphyromonas gingivalis*	−1.99303	**0.00171**	0.07221
*Porphyromonas asaccharolytica*	−1.10914	**0.00682**	0.09992
*Porphyromonas somerae*	−0.84846	**0.02929**	0.29364
*Treponema denticola*	−1.73409	**0.01410**	0.19186

^*a*^ SCFP, *Saccharomyces cerevisiae* fermentation postbiotic (Diamond V, Cedar Rapids, Iowa, USA).

^*b*^ Bolded values represent statistical significance at the 0.05 significance level (*P* < 0.05).

^*c*^ *N* = 7 (SCFP = 3; control = 4).

**TABLE 6:** Species level results of analysis of compositions of microbiomes with bias correction (ANCOM-BC) for the microbial composition of biopsy samples of stage M4 digital dermatitis (DD) lesions collected from Holstein cows supplemented with or without *Saccharomyces cerevisiae* fermentation postbiotic (SCFP)^*a*^^,*b*,*c*^

Species	Log fold change	*P* value	Adjusted *P* value
*Desulfovibrio desulfuricans*	0.36621	**0.03278**	0.63708
*Fusobacterium nucleatum*	0.40731	**0.02738**	0.63708
*Fusobacterium pseudoperiodonticum*	−0.78145	**0.01987**	0.62238
*Brucella pseudintermedia*	0.40731	0.05631	0.81067
*Psychrobacter sp. van23A*	−1.05321	**0.00296**	0.30719
*Cutibacterium acnes*	1.25053	**0.00537**	0.33396
*Rothia dentocariosa*	0.46523	**0.01850**	0.62238
*Rothia mucilaginosa*	0.56425	0.09183	0.81067
*Glutamicibacter arilaitensis*	0.71053	0.05522	0.81067
*Flaviflexus ciconiae*	1.12847	**0.00284**	0.30719
*Corynebacterium marinum*	0.43223	0.08805	0.81067
*Catenibacterium sp. co 0103*	−0.57449	**0.02648**	0.63708
*Solobacterium moorei*	−0.68774	**0.03606**	0.65974
*Staphylococcus epidermidis*	0.69402	0.08350	0.81067
*Jeotgalicoccus sp. ATCC 8456*	0.48725	0.05256	0.81067
*Jeotgalicoccus saudimassiliensis*	0.74166	0.07572	0.81067
*Bacillus paranthracis*	0.19276	0.07646	0.81067
*Alkalibacterium putridalgicola*	0.30829	0.06561	0.81067
*Trichococcus flocculiformis*	0.36621	0.06010	0.81067
*Granulicatella elegans*	0.64564	0.07541	0.81067
*Lactococcus raffinolactis*	0.46523	**0.00742**	0.38485
*Coprococcus catus*	−0.65623	**0.00072**	0.22318
*Blautia producta*	−0.51689	**0.02972**	0.63708
*Symbiobacterium thermophilum*	0.36621	**0.03278**	0.63708
*Acutalibacter muris*	−0.58290	**0.02290**	0.63708
*Thermoclostridium stercorarium*	0.36621	0.08,67	0.81067
*Flavonifractor plautii*	−0.48701	0.06065	0.81067
*Acidilutibacter cellobiosedens*	−0.56776	0.09051	0.81067
*Anaerococcus prevotii*	−0.50457	0.05909	0.81067
*Prevotella bivia*	−0.81867	**0.02001**	0.62238

^*a*^ SCFP, *Saccharomyces cerevisiae* fermentation postbiotic (Diamond V, Cedar Rapids, Iowa, USA).

^*b*^ Bolded values represent statistical significance at the 0.05 significance level (*P* < 0.05).

^*c*^ *N* = 13 (SCFP = 7; control = 6).

### Antimicrobial resistance

**Fig 5:**
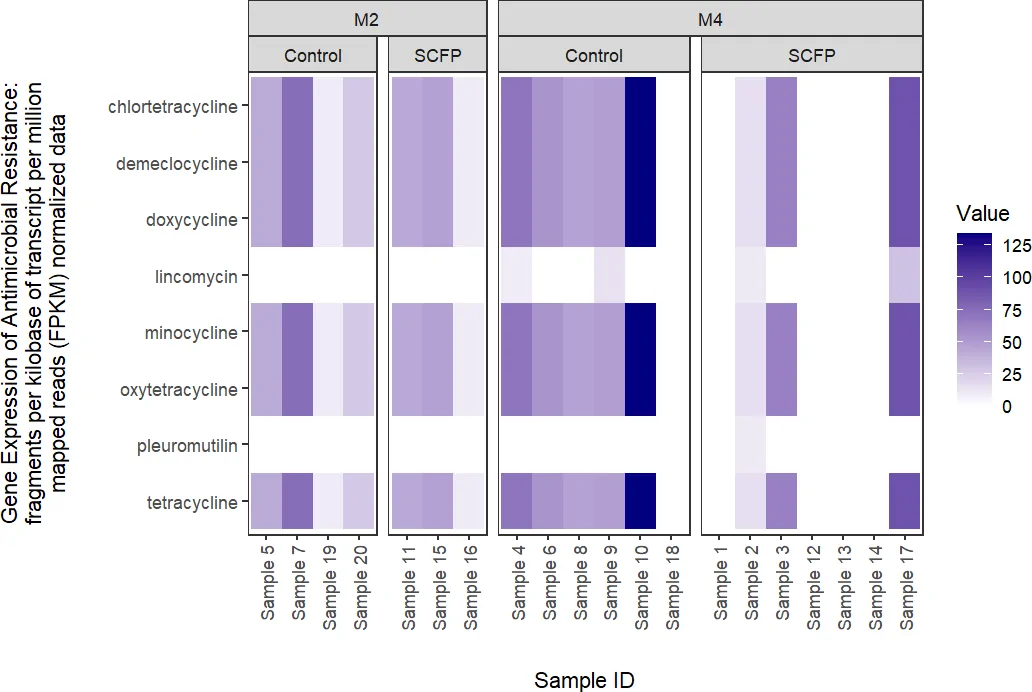
Heatmap depicting the gene expression of antimicrobial drug resistance, using fragments per kilobase of transcript per million mapped reads (FPKM) normalized data, for biopsy samples of digital dermatitis (DD) lesions collected from Holstein cows supplemented with or without *Saccharomyces cerevisiae* fermentation postbiotic (SCFP), faceted by treatment group and M-stage. M-stages: M2 lesion (an active lesion ≥ 2 cm in diameter) and M4 lesion (a chronic hyperkeratotic lesion without proliferation). *N* = 20 (*N* = 10 per treatment group).

The abundance of antimicrobial drug resistance, using FPKM normalized data, for all samples is shown in Fig. 5. Resistance was detected against chlortetracycline, demeclocycline, doxycycline, lincomycin, minocycline, oxytetracycline, pleuromutilin, and tetracycline for both the SCFP treatment and control groups. Wilcoxon rank sum testing for each individual antibiotic revealed no significant differences in antibiotic resistance between treatment groups.

## DISCUSSION

Although the complete etiology of DD remains under investigation, strong associations have been established with bacteria from the genus *Treponema* and several other bacterial species (36–42). The bacterial composition of DD lesions has also been observed to change significantly over time as the lesion develops (38–40). The constantly shifting and highly diverse bacterial composition of DD hinders the host’s immune system from developing a strong and targeted response against the disease (39–42). Previous studies have demonstrated that SCFP was associated with immunomodulating properties and reduced rates of active DD lesion development, however, the effect on the microenvironment of DD lesions is mainly unknown (31–35). Results from this study suggest that SCFP supplementation was associated with potential differences in the microbial diversity and composition of DD lesions between Holstein cows supplemented with and without SCFP, warranting further investigation. This study additionally aimed to further investigate the presence of antimicrobial resistance genes within organisms associated with DD. This was accomplished through metagenomic shotgun sequencing, which provided a complete genomic DNA analysis for all organisms present in the samples and their antimicrobial resistance factors. The identification of ARGs for antibiotics within both the tetracycline and lincosamide drug classes is consistent with the findings of (23, 23). Years of anecdotal reports within the dairy industry have suggested the possible emergence of antimicrobial resistance against the most common antibiotic DD treatments, notably tetracyclines (22). Identification of ARGs against tetracycline within DD lesions highlights a connection between untreatable, recurrent cases of DD and the overwhelming use of tetracycline-based antimicrobials as a primary treatment against the disease. Such findings have a strong clinical importance in the treatment of DD in dairy cattle that should be carefully considered.

Interpretation of the results of this study, it is important to consider the small sample size (*N* = 10 biopsies per treatment group) limits statistical power and may reduce the generalizability of the findings to the broader dairy cattle population. However, the metagenomic analyses performed in this study provide valuable, exploratory and hypothesis-generating insights into the effects of SCFP supplementation on the microbial communities of DD lesions that should be further investigated. An additional important consideration is that all samples were collected exclusively from M2 and M4 DD lesions. No healthy (M0) lesions were included, as previous attempts to sequence M0 lesion biopsies did not yield sufficient microbial reads to support comparative analysis. Consequently, the data set represents only DD affected feet, either active (M2) or chronic (M4) lesions, and cannot be directly compared with healthy feet, as in previous studies (38–41).

### Microbial diversity

Results of the alpha diversity analysis in this study found no significant differences between treatment groups with either M2 or M4 lesions and no significant differences between pens of the same treatment group when using the Chao1, Shannon, and Inverse Simpson indices. This indicated that upon development of a DD lesion (M2 or M4), SCFP supplementation did not alter the richness or evenness of the microbial species identified within the lesion. Similarly, there was no statistically significant difference in beta diversity between treatment groups with either M2 or M4 lesions and no significant differences between pens of the same treatment group as assessed by Bray-Curtis dissimilarity. However, PERMANOVA testing found a trend for potential differences in beta diversity between SCFP-supplemented and control cows with M4 DD lesions (*P* = 0.051). PERMDISP testing of M4 lesions between treatment groups found no differences, indicating the trend for potential differences in microbial composition was not driven by within-group variability. While this difference was only a potential trend associated with SCFP supplementation, this could indicate that variations in management practices, such as diet, may contribute to the variation in microbial species depicted in DD lesions of different studies (38–42).

### Microbial composition

The microbial composition of DD lesions in this study was overwhelmingly comprised of *Treponema* species, which was consistent with multiple studies investigating the primary etiology of DD (36–40). This study also found a notable prevalence of other species, including *Bacteroides spp*., *Campylobacter spp*., *Dichelobacter spp*., and *Porphyromonas spp*., which have been noted in other studies to be associated with the development of DD (38–42). In Fig. 3 and 4, samples 1 and 2 visually appeared to lack such a prominent relative abundance for *Treponema* species. This difference highlights the complexity of microbial populations within DD lesions throughout various stages of development and the need for further research.

Upon investigation into the differences in microbial composition between cows supplemented with or without SCFP, this study identified multiple significant differences in the abundance of specific microorganisms between treatment groups with either M2 or M4 DD lesions. Active M2 DD lesions in SCFP-supplemented cows were found to have significantly lower abundance of the genera *Desulfovibrio*, *Pseudomonas*, *Staphylococcus*, *Anaerotignum*, *Caproicibacterium*, and *Bacteroides*. More specifically, at the species level, SCFP-supplemented M2 lesions had significantly lower abundance of the species *Desulfovibrio sp. G11*, *Anaerotignum sp. MB30-C6*, *Caproicibacterium argilliputei*, and *Prevotella intermedia*. In contrast, M2 lesions of SCFP-supplemented cows were found to have significantly higher abundance of the genera *Fusobacterium*, *Citricoccus*, *Listeria*, and *Fundicoccus*. More specifically, at the species level, SCFP-supplemented M2 lesions had significantly higher abundance of the species *Fundicoccus culcitae* and *Helcococcus ovis*. Chronic M4 DD lesions in SCFP-supplemented cows were found to have significantly low abundance of the genera *Blautia* and *Petrimonas*. No differences were found between treatment groups for M4 lesions at the species level. These taxa represent a wide range of bacteria with various characteristics such as aerobic/anaerobic status, Gram classification, inflammatory potential, opportunistic behavior, and host versus environmental association, among other characteristics. Further investigation is needed to determine clear patterns in these microbial changes; however, this study may provide potential areas of focus.

One area that should be further investigated is the pro-inflammatory effects associated with shifts in microbial populations. *Fusobacterium* and *Helcococcus ovis* have been associated with bovine interdigital skin and mammary gland inflammation, respectively (72, 73). Elevated abundance of these taxa may suggest that SCFP supplementation is associated with a pro-inflammatory environment upon active (M2) DD lesion development. Such a finding is supported by Henige et al., which found that SCFP-supplemented cattle had a faster increase in IL-1β production upon transition from M0 DD lesion to M2 DD lesion when compared with control cattle (31).

Another area of focus should be the presence of secondary opportunistic microbes that have been identified in cases of bovine DD. This study did not find any differences in the presence of *Treponema* species, the primary pathogen associated with DD etiology, but changes in secondary pathogens may reveal broader changes in bovine health and more nuanced alterations in the development of DD. For example, *Bacteroides* and *Fusobacterium*, two genera that were found to be altered by SCFP supplementation in this study, are known to inhabit the bovine gastrointestinal tract (74, 75). Changes in their abundance could suggest that altered gut microbiomes, associated with dietary supplementation, have wider implications for bovine health that ultimately affect the development of more specific diseases such as DD. Future studies tracking changes in secondary pathogens may also uncover more clear patterns such as preferences toward bacteria with specific environmental preferences that could highlight the potential effect SCFP supplementation may have on the microenvironment of bovine digital skin.

### Antimicrobial resistance

Results of this study found ARGs associated with antimicrobials belonging to three drug classes: lincosamide (lincomycin), pleuromutilin (pleuromutilin), and tetracycline (chlortetracycline, demeclocycline, doxycycline, minocycline, oxytetracycline, and tetracycline). The presence of ARGs associated with numerous tetracycline antibiotics and a lincosamide antibiotic is consistent with findings from Beyi et al. (23). Resistance to pleuromutilin in a case of DD has not been reported prior to this study, though such resistance has been observed in both human and livestock-associated environments (76). Pleuromutilin is primarily used to treat pneumonia and skin infections and has a similar mechanism of action with macrolide and lincosamide antibiotics (76, 77). The detection of pleuromutilin resistance in this study may be attributed to the similarity to lincosamide antibiotics and the frequent use of the drug to treat skin infections in livestock. This additional evidence confirming the presence of ARGs against common antimicrobials used to treat DD in biopsies of DD lesions further amplifies the concern for emergence of antimicrobial resistance in cases of DD. The clinical implications of such findings are extremely important and should be carefully considered during the treatment of bovine DD.

### Conclusions

Results of this study reveal SCFP supplementation has an association with distinct microbial populations found within DD lesions. While the small sample size limits the statistical strength and generalizability of the findings, this study highlights the potential for SCFP supplementation to alter the microenvironment of DD lesions at various stages of development and could serve as a promising alternative to antimicrobials in the prevention and treatment of bovine DD. Published literature suggests that SCFP supplementation may protect against the development of DD through multiple mechanisms, including immunomodulation, changes in microbial populations, a decreased prevalence of lesions, and delayed onset of DD outbreaks (31, 34, 35). Studies with increased sample sizes subjected to metagenomic sequencing and the collection of DD lesions belonging to more M-stages of development should be conducted to further quantify associations between SCFP supplementation and changes in microbial populations within DD lesions.

This study also identifies antimicrobial resistance genes conferring resistance to multiple antibiotics in samples from both the SCFP-supplemented and control groups. Such findings support previous studies and years of anecdotal evidence suggesting the emergence of antimicrobial resistance in bovine DD. The results of the current study may have major implications for clinical management and treatment of DD in cattle and should be carefully considered. In addition, future studies should aim to characterize the presence of specific resistance genes and their association with commonly used antibiotics for the treatment of DD. Informed topical treatment of DD along with dietary intervention, such as SCFP, has the potential to reduce the use of antimicrobials, emergence of antimicrobial resistance, and promote improved antimicrobial stewardship.

## Data Availability

Data may be available from the corresponding author upon reasonable request and subject to the terms of the study agreement.
